# Conductive Bio-Harvesting Tonic (CBT) with an Anti-Dandruff Effect Enhances Hair Growth by Utilizing Naturally Generated Electric Energy during Human Activities

**DOI:** 10.4014/jmb.2408.08014

**Published:** 2024-09-06

**Authors:** Jisun Kim, Yoonsuk Lee, Jungbum Kim, Chai Won Park, Hyeonhui Song, Jinkee Hong, Sangmin Lee, Won Hee Jung, Joo-Hyun Hong, Ki Hyun Kim, Wonhwa Lee

**Affiliations:** 1 Department of Chemistry, Sungkyunkwan University, Suwon 16419, Republic of Korea; 2 Barunbio Inc., Seoul 03722, Republic of Korea; 3 School of Chemical & Biomolecular Engineering, Yonsei University, Seoul 03722, Republic of Korea; 4 School of Mechanical Engineering, Chung-Ang University, Seoul 06974, Republic of Korea; 5 Department of Systems Biotechnology, Chung-Ang University, Anseong 17546, Republic of Korea; 6 School of Pharmacy, Sungkyunkwan University, Suwon 16419, Republic of Korea

**Keywords:** Alopecia, hair follicle regeneration, electrical stimulation, 6-pentyl-α-pyrone, anti-dandruff

## Abstract

Alopecia, while not life-threatening, significantly impacts mental health, identity, and self-esteem of those afflicted. Current pharmacological and surgical treatments often have side effects and are limited in their ability to regenerate hair follicles (HF). Therefore, effective solutions for alopecia remain elusive. We developed an innovative hair tonic capable of stimulating HF regeneration by harnessing abandoned electric energy generated during human activities, such as the frictional electric field from walking and the electric fields from electronic devices. We devised a convenient, non-volatile, and conductive hair tonic to capture these naturally occurring electric fields. We identified 6-pentyl-α-pyrone (6PP) from *Trichoderma gamsii* as an antifungal agent effective against the dandruff-associated fungus *Malassezia* that can influence alopecia and adopted it into our conductive bio-harvesting tonic (CBT). Testing on hair follicle dermal papilla cells (HFDPC) and SKH1 mice showed that CBT significantly enhanced HF proliferation and increased growth factors *in vitro* and *in vivo*. In SKH1 mice, application of CBT under electric stimulation visibly increased hair shaft length and follicle counts. Additionally, tests on actual human hair follicles demonstrated delayed hair follicle regression when electric stimulation and 6PP were applied. In conclusion, our innovative CBT offers a promising and convenient approach for improving hair growth and combating alopecia.

## Introduction

Alopecia, or hair loss, is a prevalent symptom with diverse etiologies, potentially affecting individuals at least once in their lifetime [1]. Those afflicted with alopecia often encounter diminished self-esteem, hinderance of social interaction, and depression, adversely impacting their quality of life [2-4]. There are several distinct types of alopecia, classified by their underlying causes: alopecia areata (autoimmune), androgenetic alopecia (hereditary), telogen effluvium (medical events, nutritional deficiencies, or medications), anagen effluvium (chemotherapy), tinea capitis (fungal infection), and cicatricial alopecia (scarring), among others [5]. The treatment approaches for these conditions vary depending on the type [6]. However, stimulating hair follicle dermal papilla cells (HFDPC) to promote hair growth presents a potentially effective strategy for mitigating hair loss [7-9].

In prior research, we created an electric stimulation (ES) system for hair follicles, a promising solution for alopecia, utilizing alternating current generated by human activity [10]. While this ES system demonstrated efficacy, it required users to wear accessories such as caps, patches, or hairpins, which posed limitations and inconvenience [11]. To address these issues, we developed a conductive bio-harvesting tonic (CBT), designed to concentrate electric fields directly on the scalp without any other support, thereby enhancing hair growth more efficiently. CBT can be used independently or synergistically with the ES wearable accessories to promote hair growth. Simply spraying the CBT directly to the hair roots can harvest body-mediated electric fields and produce the desired effect for up to 30 minutes. Additionally, the presence of sweat and increased body heat from physical activity can facilitate the growth of *Malassezia* on the human scalp [12]. *Malassezia* is the fungus mainly found on the human skin and comprised of 18 species, and is associated with various skin diseases including dandruff, seborrheic dermatitis, and atopic dermatitis [13-16]. Furthermore, several studies suggest that an increased presence of *Malassezia* species may contribute to hair loss [17-20]. The use of wearable accessories for ES can result in the accumulation of sweat and heat or induce excessive friction on the scalp, potentially leading to hair loss. CBT can be used to overcome those limitations.

6-Pentyl-α-pyrone (6PP) is a naturally occurring lactone molecule, found in peaches and biosynthesized by *Trichoderma* species [21]. It is renowned for its distinct coconut aroma and notable anti-fungal properties [22-24]. Our research indicates that 6PP also exhibits antifungal activity against *Malassezia* species [25, 26]. We incorporated 6PP into our CBT, which also includes ingredients that enhance conductivity and promote hair growth. Sodium PCA, a natural moisturizing factor, is widely used as a humectant in cosmetic formulations [27]. It is a naturally occurring substance in human skin and is deemed safe even at high concentrations [28]. Instead of relying on metallic or potentially harmful substances, the formulation employed sodium PCA as an electrolyte to impart conductivity. Thereby creating a comprehensive solution for both alopecia and dandruff.

CBT was tested on hair follicle dermal papilla cells (HFDPC) and SKH1 mice under conditions that simulated the electric field generated by human activity at intensity akin to those produced during running [10, 29]. The combination of the CBT and 6PP effectively concentrated the electric field in the targeted area, resulting in enhanced proliferation of HFDPCs. Furthermore, elevated concentrations of growth factors were detected in both *in vitro* and *in vivo* settings. From the CBT applied SKH1 mice, increase in hair shaft length and counts were visible to the naked eye. Also, skin tissue sections showed an increased quantity of hair follicles. Tested on the human hair follicles, applying CBT with ES successfully delayed hair follicle regression. However, application of ES, CBT, and 6PP had no significant toxicity to organs and immune cells of SKH1 mice.

## Materials and Methods

### Experimental Procedures for Chemical Analysis

Nuclear magnetic resonance (NMR) spectra were acquired using a Bruker Avance III HD 500 NMR spectrometer at 500 MHz (^1^H), with chemical shifts determined in ppm (δ). Preparative HPLC was performed using a Waters 1525 Binary HPLC pump equipped with a Waters 996 photodiode array detector (Waters Corp., USA). LC–MS analysis was conducted on an Agilent 1200 Series HPLC system (Agilent Technologies, USA) equipped with a diode array detector and a 6130 Series ESI mass spectrometer, using an analytical Kinetex C18 Å column (100 mm × 2.1 mm, 5 μm, Phenomenex) at a flow rate of 0.3 ml/min. Spots were detected on thin-layer chromatography (TLC) plates under UV light or upon heating after spraying with anisaldehyde–sulfuric acid. Column chromatography was carried out using silica gel 60 (230–400 mesh, Merck, Germany). Merck precoated silica gel F_254_ plates and RP-18 F_254s_ plates were used for TLC.

### Fungal Material and Culture

*Trichoderma gamsii* KUC1747 was obtained from the Korea University Culture Collection (KUC). The fungus was pre-cultured on potato dextrose agar (PDA) medium (Difco, USA) at 25°C for five days in the dark. Three inoculums were then transferred from the margin of a PDA plate to 5 L PDA plates and incubated at 25°C for seven days.

### Isolation of 6-Pentyl-α-Pyrone (6PP)

The cultivation of *T. gamsii* was extracted using 10 liters of MeOH to obtain the methanol (MeOH) extract. The crude MeOH extract (3.5 g) was successively applied to solvent-partition using ethyl acetate (EtOAc), and the resulting EtOAc fraction (1.2 g) was subjected to fractionation using silica gel column chromatography (diameter: 2 × 40 cm) on hexane/EtOAc gradients of 1:0, 1:100, 1:10, 1:1, and 0:1. This generated five EtOAc sub-fractions based on the TLC profiles obtained. The sub-fraction eluted using hexane/EtOAc (1:10) was further separated via chromatography using Sephadex LH-20 (diameter: 2 × 35 cm, CHCl_3_/MeOH = 1:1), a C18 column (diameter: 1 × 26 cm) using a 20%–100% MeOH gradient, and preparative HPLC (YMC J’sphere ODS-H80, 4 μm, 250 × 20 mm i.d.) at a flow rate of 4.0 ml/min in 50%–70% MeOH for 50 min, which resulted in the isolation of 6PP (50 mg).

*6PP*: Colorless oil; ESIMS *m/z*: 167.1 [M + H]^+^; ^1^H-NMR (500 MHz, CDCl_3_) δ 7.26 (1H, dd, *J* = 6.5, 9.5 Hz), 6.15 (1H, d, *J* = 9.5 Hz), 5.97 (1H, d, *J* = 6.5 Hz), 2.48 (2H, t, *J* = 7.5 Hz), 1.64–1.70 (2H, m), 1.24–1.36 (4H, m), 0.90 (3H, t, *J* = 6.5 Hz).

### Analysis of Antifungal Efficacy of 6PP

Minimum inhibitory concentration (MIC) was determined using a modified broth serial dilution method according to the CLSI (Clinical and laboratory standards institute) guideline as described earlier [30]. Total 1×10^7^ cells/ml of *Malassezia* cells were inoculated into each well of the 96 well plate containing the serially diluted 6PP. The 96 well plates were incubated at 34°C for 3 days, and MICs were determined. Ketoconazole (KTZ) was used as a reference antifungal drug for each strain.

### Cell Proliferation Assay and Sample Preparation

HFDPCs (PromoCell, Germany) were cultured using a follicle dermal papilla cell growth medium (Ready-to-use, PromoCell) supplemented with 1% penicillin-streptomycin-glutamine (PS, 100X, Thermo Fisher Scientific). Following the stabilization of HFDPCs, the cells were seeded at a density of 1 × 104 cell/well in 6-well plates and cultured for one day. Subsequently, to concentrate the electrical field on the cells consistently, electrical stimulation was performed daily to the HFDPCs for 2 h as previously described [29]. Each well was added 100 μl of CBT in the medium. Also, 6PP was treated at varying concentrations (final concentration of 10, 20, 30, 60 μg/ml) to compare its efficacy and toxicity. Cell proliferation assay was assessed over seven days using CCK-8 assay kit (CCK-8, D-PlusTM, Doingin Biotech, Republic of Korea) in accordance with individual kit manual. After stimulation, cells were harvested and lysed in RIPA buffer, followed by centrifugation at 12,000 ×*g* for 20 min to obtain protein-containing supernatants for subsequent analysis.

### Measurement of Oxygen Consumption Rate (OCR)

Oxygen consumption rate (OCR) parameters, including basal OCR, spare capacity, and maximal OCR were quantified in live cells using the Seahorse XF24 Extracellular Flux Analyzer (Agilent Technologies). As Seahorse XF24 Analyzer continuously monitors oxygen concentration and proton flux in the cell supernatant over time, these measurements are converted into OCR parameter values facilitating the direct quantification of mitochondrial respiration and glycolysis. After culturing HFDPC cells in XF24 cell culture plates for 24 h, electrical stimulation of the HDF cells was conducted according to the method outlined in the preceding section. Prior to Seahorse XF24 analysis, the culture media were exchanged with unbuffered DMEM (pH 7.4) and stabilized in a non-CO_2_ incubator to ensure precise measurement of milli-pH unit fluctuations. OCR parameters were measured under basal conditions and after injection of compounds through drug injection ports. Consequently, basic energy metabolism profiles as crucial indicators of mitochondrial function were evaluated to our test compound and through sequential addition of mitochondria perturbing agents oligomycin, FCCP, and antimycin A.

### Animal Experiments

4-Week-old male SKH1 hairless mice were obtained from Orient Bio (Republic of Korea) and used following a one-week acclimatization period. Four mice were housed per cage in an environment with controlled temperature (20–25°C) and humidity (40–45%), maintained on a 12:12-h light/dark cycle. The mice were provided a standard rodent pellet diet (Envigo) and supplied with water ad libitum. All experiment procedures complied with the guidelines of the Institutional Animal Care and Use Committee (IACUC) of Sungkyunkwan University (IACUC No.: 202111291).

### Hair Depilation

Hair depilation was conducted on the dorsal skin area of SKH1 hairless mice primarily to observe hair growth in the region subjected to electrical field by CBT and its adjacent areas. The day prior to the initiation of electrical stimulation, commercial hair removal cream was applied to the specified area for a duration of three minutes, followed by cleansing with distilled water.

### Electrical Stimulation

For inducing concentrated electrical field formation using CBT, the electrical stimulation apparatus was used as described previously [10]. The mice were subjected to electrical stimulation at 20 V for 10 minutes, three times daily at 4-hour intervals, over a period of 10 days. The electrical input source was generated by an arbitrary function generator (AFG31000, Tektronix Co.) and amplified by a linear power amplifier (PA-151, Labworks Inc.). This setup was calibrated based on the voltage induced and applied to the human body via triboelectricity caused by the motion of walking.

3.2 mg/ml 6PP extract diluent was blended with CBT by the volume ratio of 1:3 to achieve a dosage of 8 mg/kg of 6PP upon application. Prior to electrical stimulation, 200 ul of either CBT or CBT+6PP mixture was applied to a fixed region (1.3 × 1.3 cm) in the dorsal skin of the anesthetized mouse for concentrated electrical field formation.

### Electrical Measurements

The unprocessed waveform of the output voltage was measured using a digital storage oscilloscope (TBS2000, Tektronix Co.). The electric field was determined by dividing the root-mean-square (rms) voltage on the targeted scalp, located 3 mm from the CBT, by the measurement distance unit (1 mm).

### Hair Morphology Examination & Histological Analysis

Male SKH1 hairless mice underwent electrical stimulation on the dorsal area using CBT for a duration of 7 days (*n* = 4). Daily optical images were taken in the lateral view of the dorsal skin to compare the morphology of longer and denser hair. Hair follicle number and length were quantified from optical images using ImageJ version 1.54. On day 7, the outer skin from the stimulated dorsal region was dissected and washed in PBS. The skin tissue was gently dried with gauze to remove surface moisture before embedding it in OCT compound (Sakura Fintech). Samples were then cryosectioned at a thickness of 10 μm. Standard protocols were used for H&E staining and Orange G staining.

### Enzyme-Linked Immunosorbent Assay (ELISA)

Outer skin tissues were sampled on day 7. The tissues were chopped into small pieces and incubated in cold RIPA buffer overnight. Subsequently, the samples were homogenized with stainless beads and centrifuged for 20 min (12,000 ×*g*). Cell lysates were prepared as described in the previous section. Quantification of growth factors was conducted using a Profiler mouse growth factor array kit (R&D Systems, USA) according to manufacturer’s instructions. Developed films were scanned and the resulting images were analyzed using ImageJ version 1.54. The signal intensity of each sample was normalized by the positive controls. The concentrations of the growth factors FGF3 and KGF in skin lysates were measured using commercially available mouse ELISA kits (Thermo Fisher Scientific, USA), following the manufacturer's instructions.

### Western Blot

β-catenin, phosphorylated GSK3β, GSK3β, Cyclin D1, HGF, IGF-1, SCF and GAPDH were detected by immunoblotting in protein lysate from HFDPC cells exposed to electric fields. Following sodium dodecyl sulfate (SDS) polyacrylamide gel electrophoresis, immunoblotting was conducted using the following antibodies: monoclonal mouse anti-β-catenin antibody (Santa Cruz), polyclonal rabbit anti-Phospho-GSK-3β (Ser9) antibody (cell signaling technology), monoclonal mouse anti-GSK-3β antibody (cell signaling technology), monoclonal rabbit anti-Cyclin D1 antibody, monoclonal mouse anti-HGF antibody, monoclonal mouse anti-IGF1 antibody and polyclonal rabbit GAPDH antibody (Thermo Fisher Scientific).

### Complete Blood Cell Count and Clinical Chemistry

Fresh whole blood samples were used to conduct complete blood cell counts using an autohematology analyzer (Mindray, BC 5000 Vet, China). Then, plasma was separated for the assay of glutamic-oxaloacetic transaminase (GOT), Glutamic Pyruvate transaminase (GPT), blood urea nitrogen (BUN), lactate dehydrogenase (LDH) using DRI-CHEM NX500V (FUJIFILM). Each analysis was performed using the analytical instruments at the Chiral Material Core Facility Center of Sungkyunkwan University.

## Results

### Conductive Bio-Harvesting Tonic (CBT) for Hair Growth Promotion

**Fig. 1:**
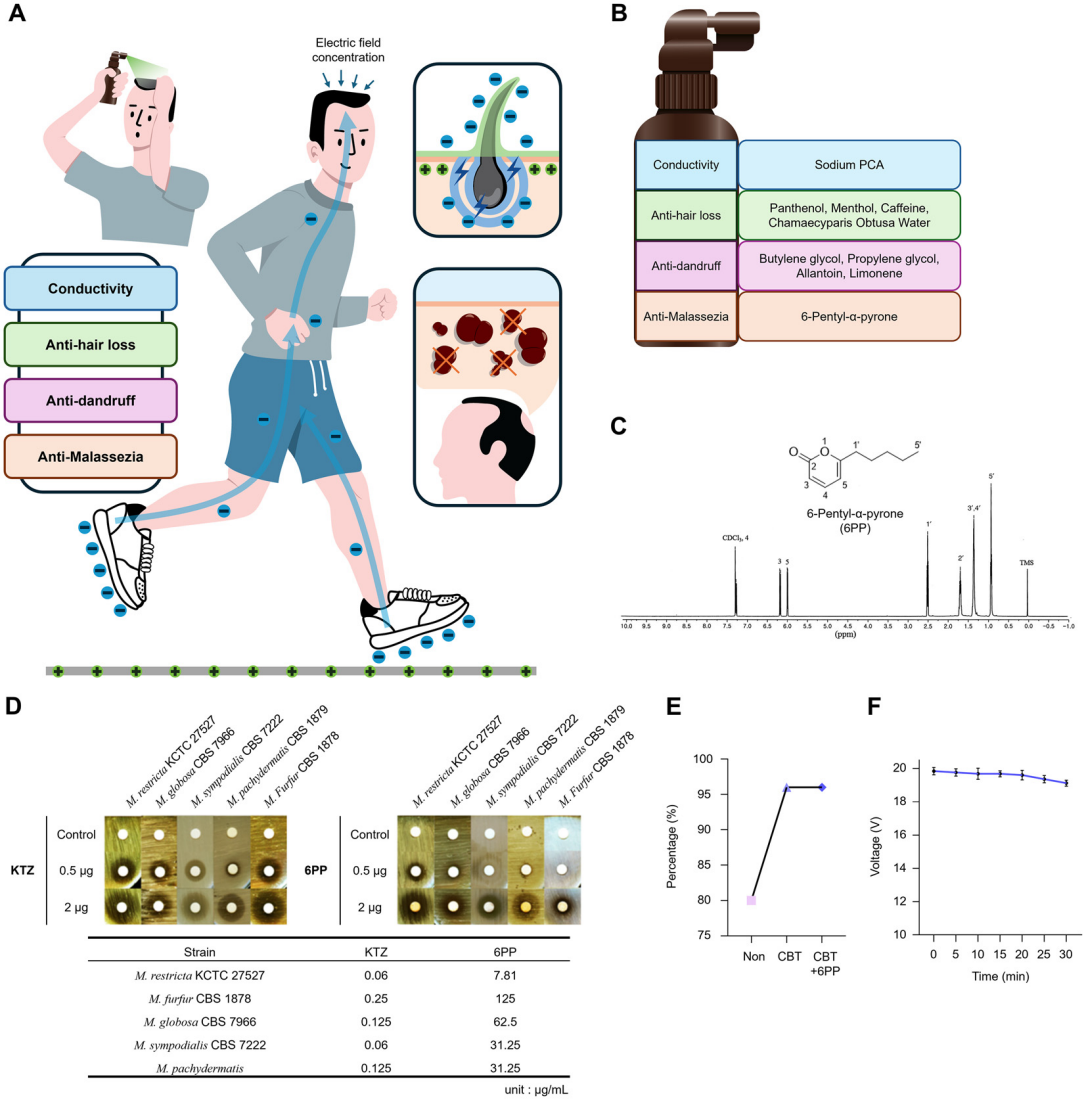
Development of conductive bio-harvesting tonic, complete solution for hair loss and dandruff. (**A**) Schematic illustration of development concept of conductive bio-harvesting tonic (CBT). (**B**) Functional design of CBT and key ingredients for the functionality. (**C**) Chemical structure of 6-pentyl-α-pyrone (6PP), the anti-dandruff agent and its 1H NMR spectrum. (**D**) Disc diffusion assay for anti-fungal activity of 6PP on *Malassezia* species (upper images), and minimal inhibitory concentrations (MIC) of 6PP against different *Malassezia* species (table). Ketoconazole (KTZ) was used as a reference standard. (**E**) Percentage of harvested electric field over the generated input, increased by application of CBT and CBT+6PP. (**F**) Time-dependent measurement of harvested voltage at the targeted site. Input: 20V, Measurements were taken 5 times at each time point.

CBT was developed, designed to harness the low-frequency alternating potential generated by human activity, concentrating it on the scalp to enhance hair growth (Fig. 1A, Table S1). It was designed to exhibit multi functionality: conductivity, anti-hair loss, anti-fungal, and anti-*Malassezia* properties (Fig. 1B). Sodium PCA, a natural moisturizing factor, acted as an electrolyte to confer conductivity to the CBT, facilitating its primary function of concentrating electric fields on the targeted area. In addition, several agents known for their anti-hair loss properties, including panthenol, menthol, caffeine, and Chamaecyparis Obtusa water, and anti-dandruff properties, such as butylene glycol, propylene glycol, allantoin, limonene were included in the formulation.

An anti-*Malassezia* compound, 6-pentyl-α-pyrone (6PP), was subsequently added (Fig. 1B). 6PP was identified from the MeOH extract of cultured *T. gamsii*, followed by purification via column chromatography and preparative HPLC. Its structure was determined by comparing its ^1^H NMR data (Fig. 1C) with previously reported data [31], as well as by analyzing the LC-MS data, which showed the molecular ion peak at *m/z* 167.1 [M+H]^+^ (Fig. S1). Anti-fungal sensitivity assay both in liquid media (MIC determination using broth dilution assay) and on solid media (disk diffusion assay) revealed that 6PP possesses anti-fungal efficacy against *Malassezia* species including *M. restricta*, which is the most predominantly observed fungal species on the human scalp, comparable to that of Ketoconazole (Fig. 1D). As mentioned earlier, abundance of *Malassezia* on calvaria of individuals with alopecia was significantly higher than that of the healthy control group. Furthermore, *Malassezia* showed a positive correlation with the incidence of androgenic alopecia [18]. Taken together, anti-*Malassezia* activity of 6PP likely paly a beneficial role against alopecia. Application of both the standard CBT and the CBT augmented with 6PP achieved a 96% concentration of the generated electric field on the desired area (Fig. 1E). The applied CBT effectively concentrated over 90% of the generated electric field for a duration of up to 30 minutes (Fig. 1F).

### Stimulation and Viability of Hair Follicle Dermal Papilla Cells (HFDPCs) by CBT with ES

**Fig. 2:**
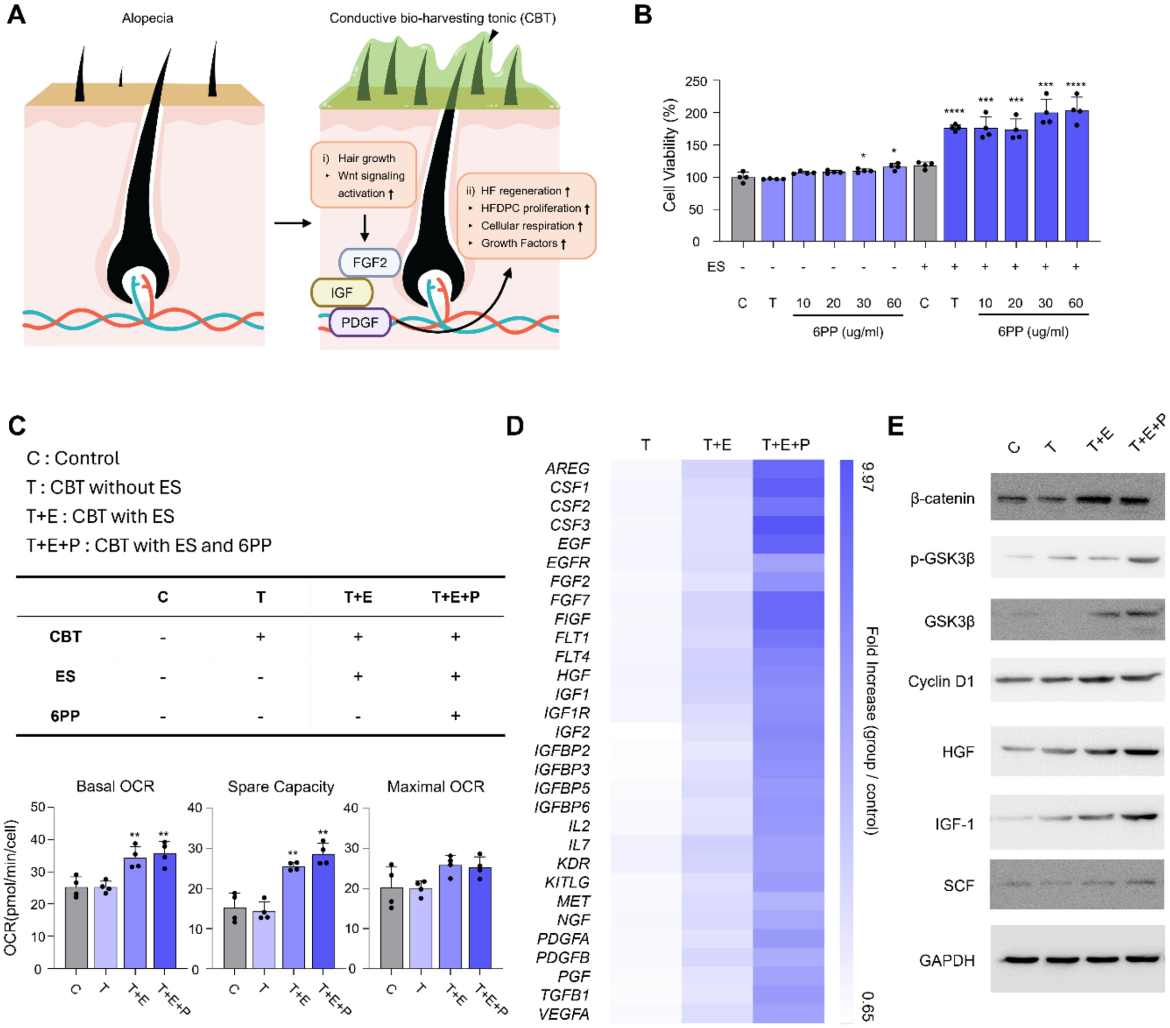
CBT promotes hair follicle dermal papilla cell proliferation. (**A**) Schematic representation of the mechanism by which CBT promotes hair growth. (**B**) Cell viability analysis of HFDPC in response to ES and escalating concentrations of 6PP. (**C**) Measurement of oxygen consumption rate (OCR) for enhanced mitochondrial activity of HFDPC. (**D**) Growth factor array of hair growth related growth factors. (**E**) The protein expression levels of GSK3β, p-GSK3β, β-catenin, Cyclin D1, HGF, IGF-1, and SCF by western blot analysis. Statistical analyses were performed using a two-tailed unpaired *t*-test. ****p < 0.0001, ***p < 0.001, **p < 0.01, *p < 0.05

Hair follicle dermal papilla cell (HFDPC) is a major compartment of the dermal layer in hair follicle, acting as an activator of hair growth cycle. HFDPCs and keratinocytes in the epidermal layer exchange signals during each phase to regulate the development of hair follicle [32]. Furthermore, The Wnt/β-catenin signaling pathway in HFDPCs contributes to determine the phenotype of follicular stem cells and morphogenesis of hair follicle [33]. Thus, the viability and cellular function of HFDPC are critical to induce early initiation and extension of growth period in the cycle (Fig. 2A). Under ES condition, the application of CBT improved the viability of HFDPCs. Also increasing the concentration of 6PP did not impede the proliferation of HFDPCs (Fig. 2B). Mitochondrial activity of HFDPCs was also enhanced by CBT+ES, with the presence of 6PP showing no adverse effects (Fig. 2C). These findings suggest that 6PP does not impede the activity of CBT+ES on HFDPCs while eradicating *Malassezia*. In addition, growth factor array results further demonstrated hair growth promoting effect of CBT+ES by activating growth factors associated with hair growth, such as FGF, IGF, and EGF (Fig. 2D) [34-36]. Moreover, CBT+ES successfully activated the Wnt signaling pathway in both groups treated with the CBT and those treated with the CBT supplemented with 6PP (Fig. 2E).

### In Vivo Hair Growth Promotion in SKH1 Hairless Mice by CBT with ES

**Fig. 3:**
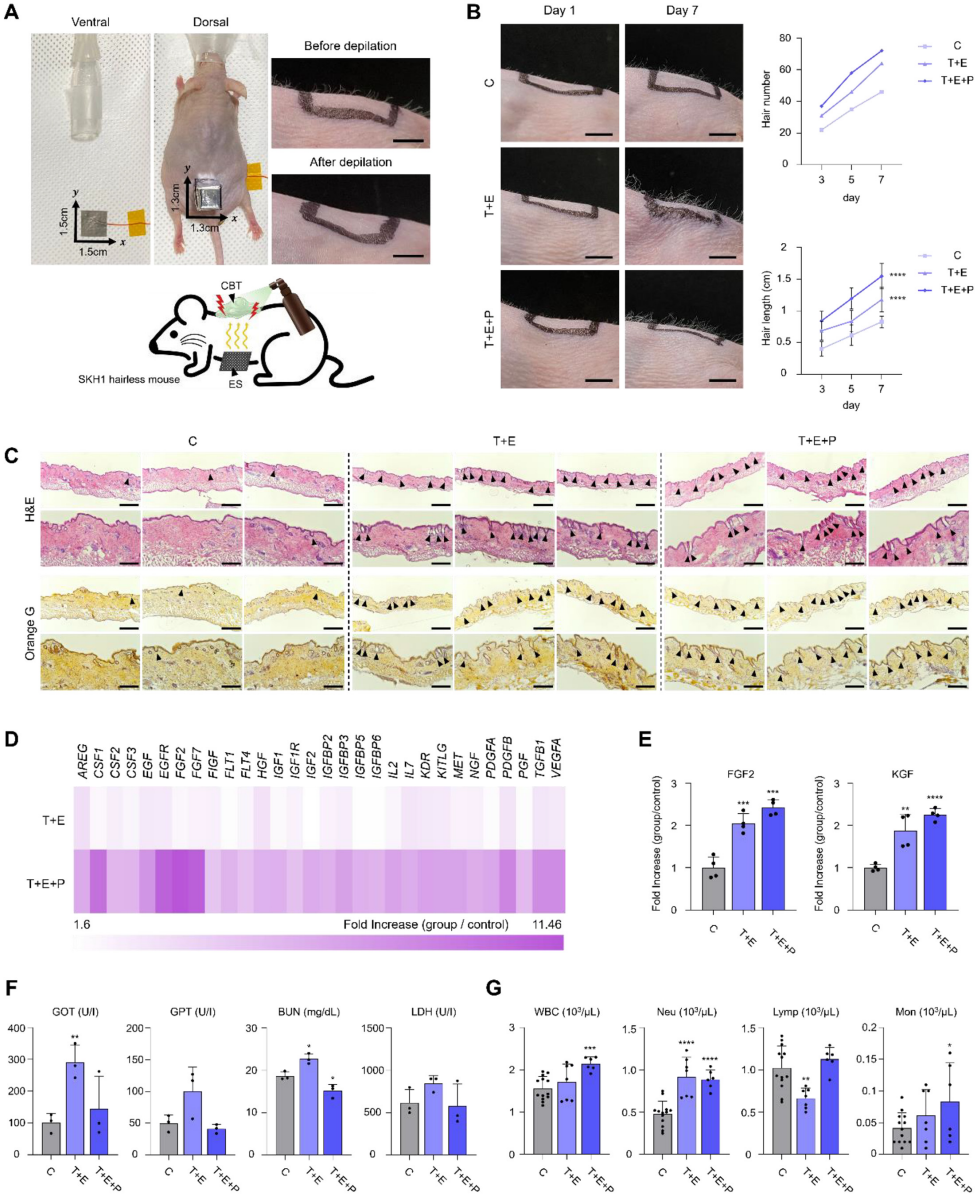
CBT induced hair growth in SKH1 hairless mouse. (**A**) Image and schematic illustration of human-activity generated electric field mimicked stimulation system. All SKH1 mice were depleted before stimulation. (right, scale: 0.5 cm) (**B**) Graphs and images of hair number and hair length of CBT applied mice. (left, scale: 0.5 cm) (**C**) Histological analysis of CBT applied mice. Hematoxylin & Eosin and orange G staining was conducted. Active hair follicles were indicated with black arrowhead. (scale: 100 μm) (**D**) Growth factor array of CBT applied mice skin tissue. (**E**) ELISA fold increase of FGF2 and KGF in skin tissue. (**F**) Organ damage marker analysis. GOT: glutamic oxaloacetic transaminase, GPT: glutamic pyruvic transaminase, BUN: blood urea nitrogen, LDH: lactate dehydrogenase (**G**) Complete blood count results. WBC: white blood cell, Neu: neutrophil, Lymp: lymphocyte, Mon: monocyte. Statistical analyses were performed using a two-tailed unpaired ttest. ****p < 0.0001, ***p < 0.001, **p < 0.01, *p < 0.05

We established an ES setup that mimicked the human activity generated electric field profile. In SKH1 mice, hair was entirely depleted using a depilatory cream, followed by ES and CBT treatment over a period of 7 days (Fig. 3A). In the group subjected to CBT+ES, a greater number of hair shafts were visibly observed, and the hair length exceeded that of the control group (Fig. 3B). Under histological analysis, newly formed hair follicles were observed in the groups applied with CBT+ES, whereas no new hair follicles were detected in the control group (Fig. 3C). In the SKH1 skin tissue, growth factor array showed increased amounts of growth factors, especially FGFs (Fig. 3D). The activation of FGF and KGF is known to increase β-catenin expression and stimulate the growth of HFDPCs [35]. The application of CBT+ES on SKH1 mice resulted in two-fold increase of FGF and KGF, confirmed via ELISA (Fig. 3E). In addition, blood samples were collected to assess organ damage markers (Fig. 3F), and blood cell counts (Fig. 3G). All results fell within normal ranges, indicating no hepatic, renal, hematologic damage was triggered as a result of ES or CBT. Together, applying CBT with ES encourages hair growth by upregulating the expression of growth factors and activating the Wnt signaling in vivo.

### Human Hair Follicle Regression Test and Impact of CBT with ES

**Fig. 4:**
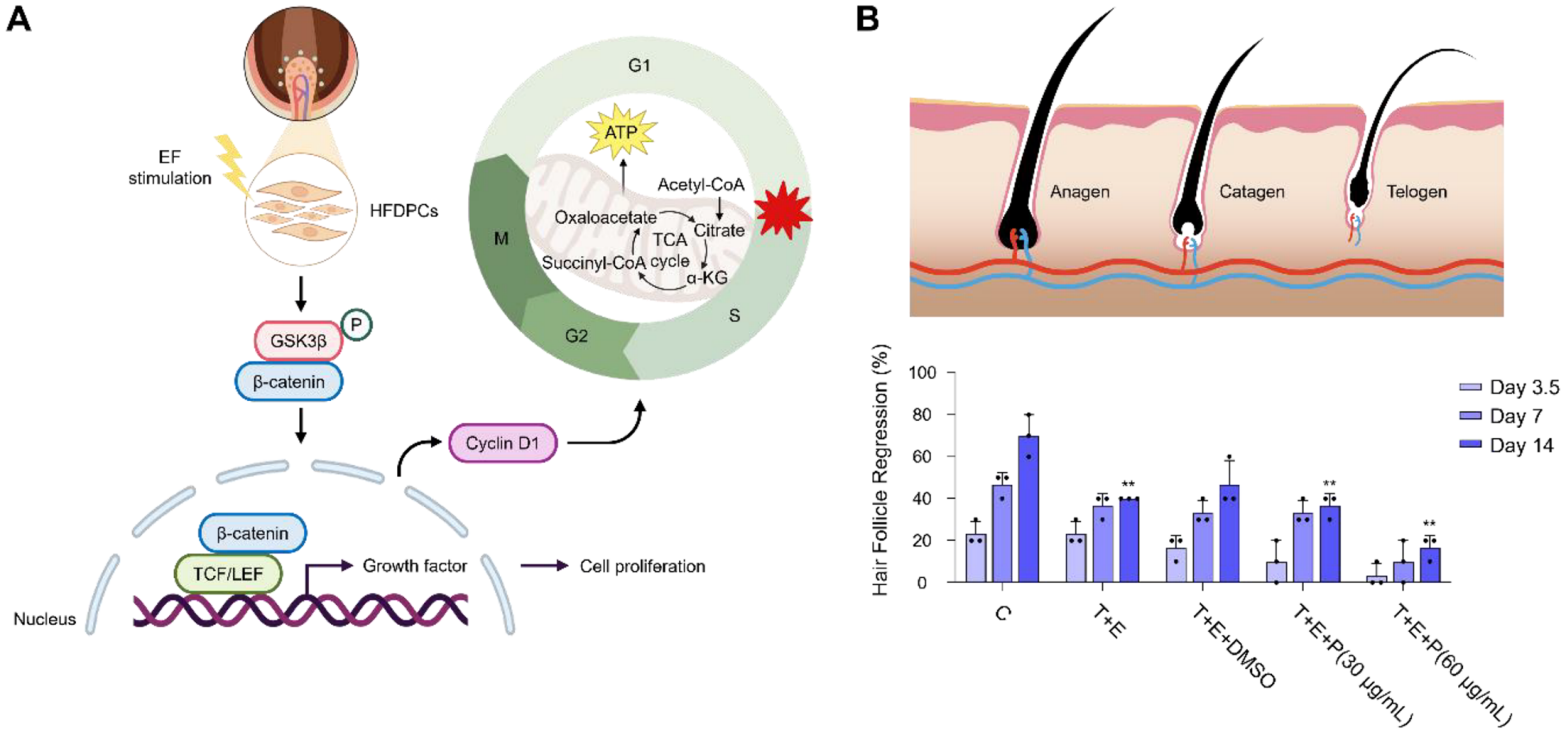
CBT delayed human hair follicle regression. (**A**) Graphical illustration of MOA of CBT. (**B**) Schematic illustration of hair follicle regression stage and ex vivo human hair follicle regression test. Statistical analyses were performed using a two-tailed unpaired *t*-test. **p < 0.01

To boost cell activity in HFDPCs through electric fields, we expected that the electrically stimulated cells are associated with activation of the Wnt/β-catenin signaling pathways, which are crucial for regulating hair cycles. The Wnt signaling pathway relies on β-catenin expression levels, which can promote hair regrowth. This study aimed to assess the impact of CBT on human HFDPCs (Fig. 4A).

By the histological analysis of CBT+ES treated SKH1 skin, the number of newly formed hair follicles were significantly higher than that of control group. While control group hair follicles were primarily in telogen phase (quiescence), CBT+ES groups were predominantly comprised of anagen phase (hair growth). Over the hair growth cycle of anagen (hair growth), catagen (transition), and telogen (quiescence), whether CBT+ES can delay hair follicle regression of entering a catagen phase has been tested on human dermal hair follicles. As a result, on day 14, the percentage of hair follicles entering the catagen phase was significantly reduced under ES conditions from 70% to 16.7% (Fig. 4B).

## Discussion

Alopecia is not a life-threatening condition; however, it is a distressing condition for the patients due to its significant impact on their mental health including sense of identity and self-esteem [37]. In this study, we aimed to determine whether the conductive bio-harvesting tonic (CBT) could promote hair growth under an electric stimulation condition. Any electrical interference was observed when the CBT with 6PP was applied to the SKH1 mouse skin and the inhibitory effect of 6PP on dandruff and alopecia associated fungus *Malassezia* was demonstrated. Consistent with the previous research of evaluating the effects of ES on HFDPC, we were able to confirm that ES achieved by application of CBT can effectively increase expression of growth factors (HGF, IGF-1, SCF) and improve WNT/β-catenin signaling pathway, thereby enhancing HF function [10]. 6PP increased the expression of phosphorylation form of GSK3β which is an indicator of activation of WNT signaling pathway, however, there was no significant change in the expression of β-catenin. Furthermore, 6PP enhanced ATP synthesis in mitochondria via ES system and stimulated cell division in cell cycle [11]. Application of CBT had direct effect of increasing the length and number of hairs. Also, histological analysis showed a significantly increased number of HF in the epidermis, indicating that CBT+ES can stimulate and regenerate the formation of HF. Based on in vivo results, we tested the hypothesis that the enhanced hair regeneration and increased HF proliferation stimulated by CBT could be attributed to ES regulation of growth factor secretion. To test this hypothesis, we investigated two key growth factors, fibroblast growth factor 2 (FGF2) and keratinocyte growth factor (KGF). Fibroblast growth factor 2 (FGF2) has a hair-growth promoting effect by enhancing hair cell proliferation and keratinocyte growth factor (KGF) generated by the dermal papilla, stimulates hair matrix cells, leading them to produce keratinocytes that form the new hair shaft [38, 39]. Fibroblast growth factor 2 (Fgf2) and keratinocyte growth factor (KGF) were significantly increased by electric stimulation and high concentration of 6PP in skin tissue. It indicates that applying electric stimulation and 6PP is concerned with the increased protein expression of FGF and KGF, growth factors involved in hair growth. Furthermore, we found that hair follicle regression is delayed in the period entering a catagen phase when electric stimulation and 6PP were applied. This analysis confirmed that CBT shows beneficial effects, demonstrating the concept that CBT can be a potential adjuvant to enhance alopecia as a HF stimulator.

Currently, the primary treatment methods for alopecia include pharmacological and surgical treatment [40]. Topical minoxidil and oral finasteride, which are FDA-approved pharmacological treatment for alopecia, are known to slowing hair loss by extending the anagen phase and promoting hair regrowth by enhancing both hair diameter and density [41]. Hair transplantation surgery, which is a representative surgical treatment, provides natural and aesthetically pleasing result for alopecia patients [42]. However, these therapies are still faced with challenges including side effects on human body, financial burden and inconvenience of use [41]. In the previous research, we developed a wearable ES device using human activity-driven internal hair follicle stimulation system (HIFS) to overcome existing alopecia treatment challenges, [10]. and in this study, we introduced a practical and convenient hair tonic as a new strategy for improving the limitations of using the device. We demonstrated that CBT could stimulate HF by harvesting the abandoned electric energy during exercise without help of any device. In this regard, this novel hair tonic has industrial value and it may offer advantages over existing alopecia treatments in terms of distinction and convenience. CBT has only been tested *in vitro* and *in vivo*; however, further human trials must be needed. ES is artificially generated in vivo test because mouse cannot generate electric fields on their own. Since we have seen that 6PP increases growth factors associated with hair growth *in vitro* and *in vivo*, we expect that it may have anti-dandruff and increasing hair growth-related growth factor effects, as well as wound healing effects. Furthermore, mixing 6PP with antibiotics can extend the efficacy of 6PP with the dual effect of wound antiseptic. Some studies indicate that 6PP suppresses LPS-induced nitric oxide levels and proinflammatory responses by inhibiting NF-κB nuclear localization and dephosphorylating MAPKs, thereby protecting macrophages against oxidative stress and excessive inflammation via the Nrf2/HO-1 pathway [43]. In this study, we have focused here only on 6PP’s anti-dandruff effect, a novel effect of 6PP must be studied in further research.

In summary, we identified the effects of CBT, demonstrating significant increases in HFDPC activity and HF activation. CBT and 6PP effectively enhances growth factor synthesis associated with hair growth and improves signaling pathways, thereby boosting HF functionality for hair cycle maintenance. These data support the notion that CBT represents a novel therapeutic paradigm with no side effects.

## Supplemental Materials

Supplementary data for this paper are available on-line only at http://jmb.or.kr.
