# Supplementary material for: Conductive Bio-Harvesting Tonic (CBT) with an Anti-Dandruff Effect Enhances Hair Growth by Utilizing Naturally Generated Electric Energy during Human Activities
Source: J Microbiol Biotechnol. 2024 Sep 6;34(11):2376–84. doi: 10.4014/jmb.2408.08014 (PMC11637866; doi:10.4014/jmb.2408.08014)
Supplement: Supplementary file 1 [file jmb-34-11-2376-supple.pdf]

J. Microbiol. Biotechnol. 2024. 34(11): 2376–2384

https://doi.org/10.4014/jmb.2408.08014

Supplementary Table and Figure

Table S1. Chemical formula of CBT.

Name	Full ingredient
Conductive bio-harvesting tonic	Water, Alcohol, Butylene Glycol, Propylene Glycol, Dipropylene Glycol, Polysorbate 80, Caffeine, Sodium PCA, Sorbitol, Menthol, Alanine, Chamaecyparis Obtusa Water, Panthenol, Allantoin, Ammonium Glycyrrhizate, Phenoxyethanol, Perfume, Alpha-Isomethyl Ionone, Benzyl Benzoate, Benzyl Salicylate, Limonene, Hexyl Cinnamal, Hydroxycitronellal, Linalool

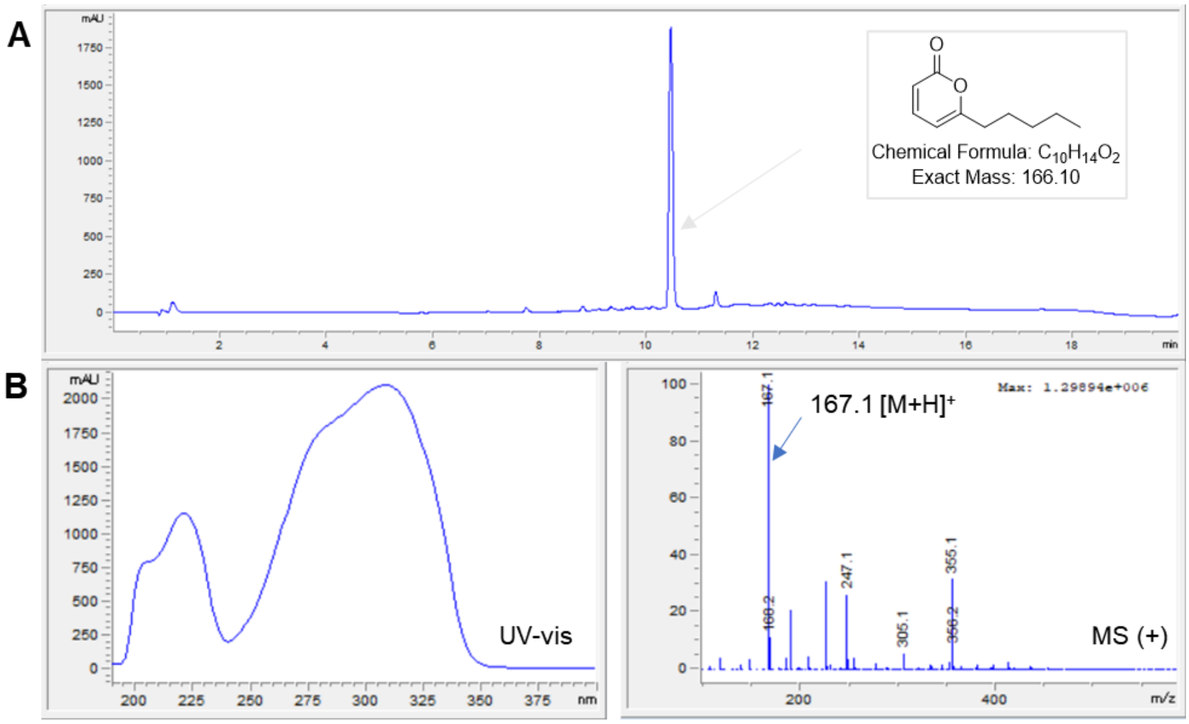

Fig. S1. (A) UV chromatogram of LC/MS (detection wavelength was set as 254 nm) and (B) UV and MS data for 6-pentyl-α-pyrone (6PP).
